# Variable Number Tandem Repeat Profiling of *Mycobacterium ulcerans* Reveals New Genotypes in Buruli Ulcer Endemic Communities in Ghana and Côte d’Ivoire

**DOI:** 10.3389/fmicb.2022.872579

**Published:** 2022-06-23

**Authors:** Elizabeth Gyamfi, Magdalene Amerl Dogbe, Charles Quaye, Abel Adjet Affouda, Edwin Kyei-Baffour, Daisy Awuku-Asante, Mabel Sarpong-Duah, Lydia Mosi

**Affiliations:** ^1^Department of Biochemistry, Cell and Molecular Biology, University of Ghana, Accra, Ghana; ^2^West African Center for Cell Biology of Infectious Pathogens, University of Ghana, Accra, Ghana; ^3^Department of Biological Sciences, Mississippi State University, Starkville, MS, United States; ^4^Noguchi Memorial Institute for Medical Research, University of Ghana, Accra, Ghana; ^5^Department of Sociology and Anthropology, University of Jean Lorougnon Guédé, Daloa, Côte d’Ivoire

**Keywords:** *Mycobacterium ulcerans*, variable number tandem repeats, genotypes, Buruli ulcer, mycolactone-producing mycobacteria

## Abstract

Buruli ulcer (BU), a necrotic skin disease caused by *Mycobacterium ulcerans*, is mainly prevalent in West Africa, but cases have also been reported in other tropical parts of the world. It is the second most common mycobacterial disease after tuberculosis in Ghana and Côte d’Ivoire. Heterogeneity among *M. ulcerans* from different geographical locations has not been clearly elucidated, and some studies seem to suggest genetic differences between *M. ulcerans* in humans and in the environment. This study aimed at identifying genetic differences among *M. ulcerans* strains between two BU endemic countries: Ghana and Côte d’Ivoire. Clinical samples consisting of swabs, fine needle aspirates, and tissue biopsies of suspected BU lesions and environmental samples (e.g., water, biofilms from plants, soil, and detrital material) were analyzed. BU cases were confirmed *via* acid fast staining and PCR targeting the 16S rRNA, IS*2404*, *IS2606*, and ER domain genes present on *M. ulcerans*. Heterogeneity among *M. ulcerans* was determined through VNTR profiling targeting 10 loci. Eleven *M. ulcerans* genotypes were identified within the clinical samples in both Ghana and Côte d’Ivoire, whiles six *M. ulcerans* genotypes were found among the environmental samples. Clinical *M. ulcerans* genotypes C, D, F, and G were common in both countries. Genotype E was unique among the Ghanaian samples, whiles genotypes A, Z, J, and K were unique to the Ivorian samples. Environmental isolates were found to be more conserved compared with the clinical isolates. Genotype W was observed only among the Ghanaian environmental samples. Genotype D was found to be prominent in both clinical and environmental samples, suggesting evidence of possible transmission of *M. ulcerans* from the environment, particularly water bodies and biofilms from aquatic plants, to humans through open lesions on the skin.

## Introduction

The causative agent of Buruli ulcer (BU), *Mycobacterium ulcerans*, is responsible for the third most common mycobacterial infection in humans. This skin disease is mostly found in the tropical and sub-tropical regions of the world, particularly in Africa and in some regions with temperate climates such as Japan, Papua New Guinea, and Southern Australia (Wagner et al., 2008; Nakanaga et al., 2011; Omansen et al., 2019). Rural areas close to swamps, wetlands, or slow-flowing rivers are the common environmental locales where the disease is endemic although the definite reservoirs remain a mystery (McIntosh et al., 2014). The disease causes extensive necrosis of the skin and underlying tissues which can lead to severe ulceration if treatment is delayed (Johnson et al., 2005). Even though BU is not fatal, the associated lengthy hospital stays during treatment, risk of secondary infections, permanent deformities and joint contractures, cost of surgery, and social stigma often result in a dire social and economic burden on the families of affected individuals (Grietens et al., 2008; Adamba and Owusu, 2011; Gyamfi et al., 2021).

The exact route of disease transmission is not clearly known. This is partly due to inadequate knowledge on the incubation period for human infection, the prolonged duration between BU symptom manifestation, and the treatment-seeking behavior of patients with BU (Williamson et al., 2012; Amoako et al., 2019). Transmission is, however, associated with aquatic environments and areas with disturbed landscapes due to mining activities, deforestation, flooding, and dam construction (Merritt et al., 2010; Williamson et al., 2012). The bacterium has also been found to be more adapted to areas with low oxygen and light (Ramakrishnan et al., 1997; Palomino et al., 1998). Human-to-human transmission of BU has not been demonstrated.

The insertional sequence element IS*2404*, which is present in over 240 copies in the genome of *M. ulcerans* (Narh et al., 2015), is the WHO-approved polymerase chain reaction (PCR) target for case confirmation of BU in clinical samples. Unfortunately, this target has been detected in different environmental matrices including water samples, soil, detritus, biofilms from aquatic plants, collected from both BU endemic and non-endemic community-associated water bodies (Johnson et al., 2005; Narh et al., 2015; Tano et al., 2017) and possum feces (Tobias et al., 2016). This insertion sequence has also been identified in other organisms such as aquatic insects (Marsollier et al., 2002; Benbow et al., 2008), and tree-dwelling ringtail and brushtail possums in Australia (Carson et al., 2014; O’Brien, 2017).

Even though IS*2404* is excellent in confirming BU in clinical samples due to the high copy number, its usage on environmental samples remains a challenge. Molecular detection of *M. ulcerans* DNA using primers designed for IS*2404* often leads to amplification of non-specific targets due to the likely presence of other mycolactone-producing mycobacteria in environmental samples that may harbor this insertion sequence, such as *Mycobacterium liflandii* and *Mycobacterium pseudoshottsi* (Narh et al., 2015). The presence of PCR inhibitors in the environmental samples also contributes to challenges with successful PCR amplification (Nakanaga et al., 2013; Tano et al., 2017). Few studies have determined the relationship between the presence of *M. ulcerans* and its occurrence in BU endemic areas (Maman et al., 2018). There is also a paucity of information on the ecology of *M. ulcerans* and how it is distributed in BU endemic areas.

Variable number tandem repeat (VNTR) discrimination of *M. ulcerans* mostly relies on the four standard loci. However, recent studies have shown a rapid genetic evolution of *M. ulcerans* and other mycolactone-producing mycobacteria (MPM) (Demangel et al., 2009; Röltgen et al., 2012). Targeting other VNTR loci could help in differentiating *M. ulcerans* and other MPMs present in both clinical and environmental samples.

This study sought to determine the relationship between the ecology of *M. ulcerans* and BU endemicity in Ghana and Côte d’Ivoire. This was necessary because some geographical locations reporting BU cases differ physically from historic *M. ulcerans*-positive locations. Identifying such discordant sites where environmental *M. ulcerans* is present but no BU cases are reported or areas reporting BU cases but with no local presence of the pathogen will be important in transmission studies and to confirm if seasonality could account for the differences in geographical patterns (Pileggi et al., 2017). The study also sought to identify the genotypes of *M. ulcerans* circulating in the environment within and between the two countries. Data from this study are essential in determining the link between clinical and environmental *M. ulcerans* strains circulating in Ghana and Côte d’Ivoire, the 2 countries most affected by BU globally.

## Materials and Methods

### Ethics Approval Statement

Ethical approvals for this study were sought from the Institutional Review Board of Noguchi Memorial Institute for Medical Research (052/17-18), Ghana Health Service Ethical Review Committee (GHS-ERC017/07/17), and the Comité National d’Ethique et de la Recherche (CNER) in Abidjan, Côte d’Ivoire (112-18/MSHP/CNESVS-km). All participants consented to be enrolled in this study. All children below the age of 18 had their parents’ and guardian’s consent before enrollment. BU case diagnosis and treatment were done at no cost to participants. All participants who were positive but were not on treatment were reported to their nearest respective health centers for BU treatment and wound care. Each participant filled a questionnaire that solicited information on the disease, mode of transmission, and treatments administered.

### Sites for Both Clinical and Environmental Samples

#### Clinical Sampling Sites

**FIGURE 1:**
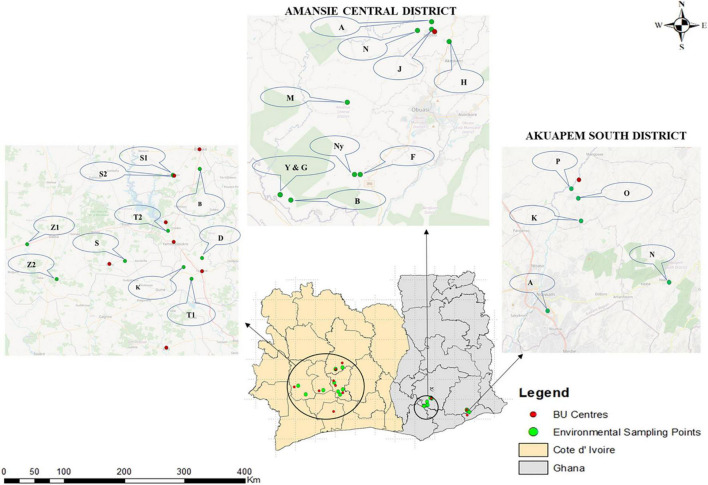
Clinical and environmental sampling sites in Ghana and Côte d’Ivoire. Fifteen communities were selected for environmental sampling from two out of the three districts where clinical sampling was done in Ghana, while 10 BU endemic communities were selected from Côte d’Ivoire. Selection for environmental sampling was done after disease confirmation from BU centers based on areas with a high number of confirmed BU cases and the presence/nearness to a source of water body used by the communities for domestic and commercial purposes based on an administered questionnaire. The environmental sampling sites for Côte d’Ivoire were Zoukougbeu [Gbeulissou-Adjoablassou (Z1) and Grebeu (Z2)], Sinfra [Djenedoufla-Gbalibonou (S), Kongouanou (Djekanou (T2) and Kongouanou (K)], Sakassou [N’zokossou (S1) and Amonlima (S2), Bouake (Adjebretti (B), Toumoudi (Angoda (T1), and Divo (Divo-Sakota (D)]. For Akuapem South District, 5 communities were selected [Pakro (P), Obosono (O), Otukwadwo (K), Alafia (A), and Pokrom (N)], and for Amansie Central, 10 communities were selected [Nkoduase (N), Aboabo (A), Jacobu (J), Homase (H), Mile eleven (M), Fenase No.2 (F), Nyamebekyere (Ny), Gyaman (G), Apaaho (b), and Yaakrakrom (Y)].

Clinical samples were obtained from health facilities serving three BU endemic district in Ghana, namely, Amasaman District Hospital in the Ga West District of Greater Accra, St Peter’s Hospital (Jacobu) in the Amansie Central District of Ashanti region, and Pakro Health Center in the Akuapim South District, and eight BU hospitals in Côte d’Ivoire (Figure 1), namely, “Centre Jean Baptiste Vatelot” in Bouaké, “Centre UB de Divo” in Divo, “Santé Notre Dame du Camel” in Sakassou, “Centre de Santé de Djenedoufla” in Sinfra, “Centre Saint Michel de Zoukougbeu” in Zoukougbeu, “Centre UB de Kongouanou” in Kongouanou, “Centre de Sainte-famille” in Yamoussoukro, and “Centre UB de Djekanou’ in Toumoudi (Djekanou).” Active case searches were also undertaken in all the selected areas.

#### Environmental Sampling Sites

Ten BU endemic communities in Amansie Central District, five communities in Akuapim South District, and eight BU endemic towns in Côte d’Ivoire (Bouaké, Divo, Sakassou, Sinfra, Zougougbeu, Kongouanou, Yamoussoukro and Djekanou) were selected for environmental sampling (Figure 1). For Côte d’Ivoire, two sampling sites were selected in Konguoanou and Zougougbeu. Priority was given to villages that had reported high numbers of confirmed BU cases during this study. All the fifteen study sites selected in Ghana are in the southern part of the country, while those in Côte d’Ivoire are located in the southern, western, and central parts of the country. The climate of all selected communities is mostly tropical. Most communities have been disturbed by either mining activities, deforestation, or flooding. Inhabitants of communities in both countries mainly rely on water bodies such as the ponds, streams, and lagoons or boreholes within the communities for both domestic and economic activities such as washing, cooking, farming, and fishing, as well as recreational activities like swimming.

### Clinical and Environmental Sample Collection

This cross-sectional study aimed at comparing *M. ulcerans* genotypes between clinical and environmental samples collected from Ghana and Côte d’Ivoire. There was a passive case identification in all selected health facilities and an active case search in communities for suspected BU cases. A cross-sectional standardized sampling was conducted in both countries to collect environmental samples.

#### Clinical Sample Collection for Buruli Ulcer Confirmation

Clinical samples were collected between November 2016 and January 2020 in both countries. Sterile cotton swabs (for ulcerative lesions) and fine-needle aspirates (for pre-ulcerative lesions) were collected by medical personnel and other trained health workers in the communities. Three specimens were collected per lesion into sterile 2 ml screw cap tubes: Two in 1 ml each of 1 × phosphate-buffered saline (for staining and PCR) and one in PANTA transport media (for culture). The samples were kept at 4°C in a refrigerator at the respective health centers until ready for transfer to the laboratory. All samples were transported *via* cold chain.

#### Environmental Sample Collection for *Mycobacterium ulcerans* Confirmation

Environmental samples were collected between April and October 2019 in Ghana and July 2019 in Côte d’Ivoire. These collection periods coincided with the rainy season in both countries. Areas for sampling were selected based on a higher number of confirmed BU cases from this study and proximity to a frequently used water body by the inhabitants for domestic (bathing and cooking) or commercial purposes (farming). For water bodies, priority was given to rivers (and slow-moving streams). A pond or borehole was considered in the absence of a river.

**FIGURE 2:**
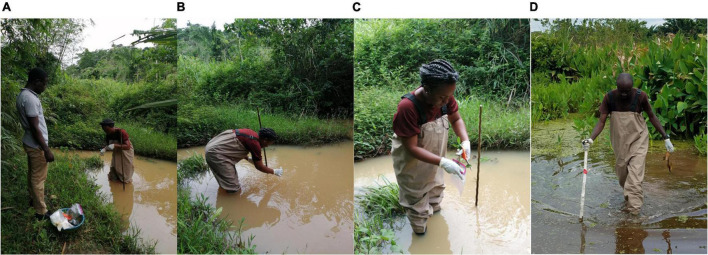
Sampling of environmental matrices used for this study. **(A)** Collection of soil samples close to a river, **(B)** water sampled from the surface of a river, **(C)** aquatic plants sampled from a river, and **(D)** detritus collected from a river.

Sampling of each water body was conducted following the protocols described by Williamson et al. (2008) and Narh et al. (2015) with some modifications and optimizations. At each water body, four matrices were collected in duplicates (i.e., water residue, biofilms from aquatic plants, detritus, and soil). For water residue, a sterile water bottle was used to scope the surface water covering an area of about 1 m^2^: one from within the river (inner-I) (Figure 2A) and one from the riparian zone (outer-O) (Figure 2B). A volume of 50 ml of this was poured into a 50 ml falcon tube.

For biofilm sampling, the stems and leaves of some dominant aquatic plants such as *Pistia stariores* and *Nymphaea capensis* were cut and placed into a Ziploc bag containing 100 ml of double-distilled water (Figure 2C). The bags were sealed airtight, and the biofilms were dislodged by shaking and rubbing the bag vigorously several times. A volume of 50 ml of resulting mixtures was then poured into individual 50 ml falcon tubes for storage.

For soil, a sterile spoon was used to pick about 4,000 mg (2×), one from the water floor (inner) and one from the riparian zone (outer-O). The soil samples were preserved in a 15 ml falcon tube (BD Biosciences) containing 10 ml of absolute ethanol. A method similar was used for detritus sampling, this time using a scalpel, for the collection of 10 cm long or appreciable sizes of detritus (dead plant leaves, stems, and grass blades) (Figure 2D). All matrices were well labeled and preserved on ice packs, in a cooler, in the field. Samples were later transported to the laboratory and preserved at 4°C in a refrigerator until ready for laboratory analyses.

### Sample Processing for Laboratory Analyses

The clinical samples were processed according to Gyamfi et al. (2021). The swabs were processed for culture and DNA extraction. Briefly, the PBS solution containing the swab sticks was vortexed to dislodge microbial cells. A volume of 200 μl was then used for DNA extraction using the Quick-RNA/DNA miniprep plus (New England Biolabs). The remaining mixture was preserved at 4°C for later use.

Water samples were concentrated using an optimized protocol. Water collected in each 50 ml falcon tube was spun individually at 5,000 rpm for 15 min in a refrigerated centrifuge at 4°C (Eppendorf), after which 20 ml was slowly decanted, and the remaining volume spun again at 5,000 rpm for 5 min. This step was repeated, and the remaining 10 ml was spun at high speed, after which 8 ml was decanted off. The remaining 2 ml was then preserved at 4°C for further analysis.

Dislodged biofilm was concentrated based on an optimized protocol. Each 50 ml falcon tube containing the dislodged biofilm in 50 ml of water was spun at 5,000 rpm for 5 min in a refrigerated centrifuge at 4°C (Eppendorf). A volume of 20 ml was slowly decanted and the remainder spun again at 5,000 rpm for 5 min. This step was repeated, and the remaining 10 ml was preserved at 4°C for future use. For DNA extraction, the 10 ml was vortexed for 5 min. Each suspension was allowed to settle down for 1 min, and 1 ml of the supernatant was then pipetted into a 1.5 ml Eppendorf tube, which was further centrifuged at high speed for 5 min. A volume of 800 μl of the resulting supernatant was pipetted off and the remaining 200 μl was briefly vortexed to obtain a suspension that was stored at 4°C in a refrigerator for future use.

Detritus and soil samples preserved in 10 ml of ethanol for DNA extraction were vortexed for 5 min. A volume of 1 ml of each resulting supernatant was pipetted into a 1.5 ml Eppendorf tube and further centrifuged at high speed for 5 min. A volume of 800 μl of each supernatant was pipetted off and the remaining 200 μl was briefly vortexed to obtain a suspension.

From each 200 μl solution preserved, 10 μl was used for acid fast staining, and the rest was used for DNA extraction.

### Genotyping and Confirmation of *Mycobacterium ulcerans* DNA in Both Environmental and Clinical Samples

Extraction of DNA was performed using the Quick-DNA Fungal/Bacterial Miniprep Kit (environmental samples) and Quick-DNA/RNA Miniprep Plus (for clinical samples) following the manufacturer’s procedure (New England Biolabs). Samples were first screened for the presence of mycobacteria by the amplification of the 16S hypervariable region of mycobacteria *via* PCR (Hughes et al., 1993). All positive samples were next screened for the presence of *M. ulcerans* DNA by targeting the insertion sequences IS*2404* and IS*2606* and further for the confirmation of MPM by the amplification of the enoyl reductase (ER) domain on the plasmid responsible for making mycolactone using conventional PCR (Narh et al., 2015). Both negative and positive controls were included for each run.

All samples positive for IS*2404* for clinical samples or a combination of either IS*2404* or IS*2606* and ER for environmental samples were then genotyped *via* amplification of different loci for VNTR typing. The 10 VNTR loci targeted were Locus 6, Locus 19, MIRU 1, ST1, Locus 15, Locus 18, MIRU 9, Locus 16, Locus 33, and Locus 13 for clinical and environmental samples (Table 1). Oligonucleotide VNTR primers that amplify the VNTR region of MPM were synthesized by Sigma-Aldrich and Inqaba Biotech. PCR conditions for VNTR typing were adapted from research that targeted similar loci (Ablordey et al., 2005, 2007; Williamson et al., 2014; Narh et al., 2015; Zeukeng et al., 2021). A nested PCR for the environmental DNA was performed using the same conditions and primers, if no amplicon was initially obtained from the first PCR run. Amplicon sizes of samples and controls were separated on agarose gel and viewed on a Bio-Rad Gel Doc™ XR.

**TABLE 1:** Primers used for genotyping of *M. ulcerans* from clinical and environmental samples.

Primers	Forward (5′–3′)	Reverse (5′–3′)	Annealing Temp (°C)	Size (Bp)
				
*IS2404*	AGCGACCCCAGTGGATTGGT	CGGTGATCAAGCGTTCACGA	64	492
IS2606	AGGGCAGCGCGGTGATACGG	CAGTGGATTGGTGCCGATCGAG	64	310
16rRNA	AAAAAGCGACAAACCTACGAG	AGAGTTTGATCCTGGCTCAG	56	600
ER	GAGATCGGTCCCGACGTCTAC	GGCTTGACTGTCACGTAAG	63	476
Locus 15	GCCACCGGTCAGGTCAGGTT	TCACCAACTACGACGGCGTTC	67.5	Variable
Locus 16	CCAACGCTCCCCCAACCAT	GCTCACAGGCCTTCGCTCAGA	68	Variable
Locus 18	CCCGGAATTGCTGATCGTGTA	GGTGCGCAGACTGGGTCTTA	65.4	Variable
ST1	CTGAGGGGATTTCACGACCAG	CGCCACCCGCGGACACAGTCG	65.4	Variable
MIRU 1	GCTGGTTCATGCGTGGAAG	GCCCTCGGGAATGTGGTT	64.5	Variable
MIRU 9	GCCGAAGCCTTGTTGGACG	GGTTTCCCGCAGCATCTCG	66.4	Variable
Locus 19	CCGACGGATGAATCTGTAGGT	TGGCGACGATCGAGTCTC	64	Variable
Locus 13	CAGGTATTCCAGGAGATCAAA	GGCGACAAGGCTCGTT	59	Variable
Locus 6	GACCGTCATGTCGTTCGATCCTAGT	GACATCGAAGAGGTGTGCCGTCT	68.5	Variable
Locus 33	CAAGACTCCCACCGACAGGC	CGGATCGGCACGGTTCA	65	Variable

The expected band sizes were excised and purified using the GeneJet Gel Purification kit (Thermo Fisher Scientific). The concentration and purity of the PCR products were assessed using a Thermo Scientific NanoDrop Spectrophotometer. Purified PCR amplicons that were at or above 20 ng per 100 base pair with purity at or above 1.75 were Sanger sequenced with ABI 3730XL DNA Analyzer with their respective forward primers at the Department of Biochemistry, University of Cambridge, United Kingdom. Nucleotide repeats were identified using the Tandem Repeats Finder program (Benson, 1999). Length polymorphisms at all the VNTR loci were computed based on target loci size and repeat length from published data (Ablordey et al., 2005, 2015; Williamson et al., 2008; Narh et al., 2015). Genotypes were assigned depending on the variable number of tandem repeats found at these standard loci: Locus 6, MIRU 1, ST1, and Locus 19. Isolates that could not be genotyped using the four standard loci (due to no amplification at one of the four standard loci) were further differentiated using 6 other loci, namely, Locus 15, Locus 16, Locus 18, Locus 33, MIRU 9, and Locus 13, to *M. ulcerans* or other MPMs.

### Statistical Analysis of Data Obtained

All data obtained were recorded in Microsoft Excel 2016. All statistical analysis and graphs were analyzed with GraphPad Prism version 8.4.3. Student’s *t*-test was used for comparison of clinical and environmental samples between Ghana and Côte d’Ivoire, while chi-square test for contingency table was used for comparison of proportion with significant level set at *p* ≤ 0.05.

## Results

### Buruli Ulcer Clinical Sample Confirmation

Out of the 382 samples collected from the suspected BU cases, only 11/230 (4.7%) of the samples from Côte d’Ivoire and 5/152 (9.6%) of the samples from Ghana were positive for acid-fast bacilli, whereas 335/382 (87.7%) were positive for a mycobacterial infection *via* PCR amplification of the 16S rRNA hypervariable region. Overall, 296 (77.5%) cases tested positive for IS*2404*, 217 (56.8%) cases for IS*2606*, and 183 (47.9%) cases for the ER domain. Thus, BU positivity for clinical samples were confirmed by the amplification of IS*2404* (Gold standard for BU detection). A total of 77.5% of the clinical samples were thus, positive for BU. Table 2A summarizes BU positives identified using different BU confirmatory tools.

**TABLE 2A:** Proportions of confirmed BU cases using different BU confirmatory tool.

Location	IS*2404* n (%)	IS2606 n (%)	ER n (%)	Acid fast n (%)	16S n (%)	Total n (%)
**Ghana**						
Ga east municipal	16 (61.5)	12 (46.1)	1 (3.8)	0	20 (76.9)	26
Akuapim south municipal	39 (61.9)	21 (33.3)	19 (30.1)	1 (1.6)	44 (66.7)	63
Amansie central	49 (77.8)	21 (33.3)	15 (23.8)	4 (6.3)	55 (87.3)	63
**Côte d’Ivoire**						
Bouaké	34 (100)	24 (70.6)	24 (70.6)	0	34 (100)	34
Divo	5 (100)	5 (100)	5 (100)	0	5 (100)	5
Kongouanou	34 (94.4)	34 (94.4)	28 (77.8)	0	36 (100)	36
Sakassou	12 (66.7)	13 (72.2)	10 (55.6)	3 (16.7)	17 (94.4)	18
Sinfra	3 (100)	3 (100)	3 (100)	3 (100)	3 (100)	3
Toumoudi/djekanou	41 (85.4)	33 (68.7)	32 (66.7)	2 (4.2)	43 (89.6)	48
Yamoussoukro	18 (58.1)	16 (51.6)	14 (45.2)	3 (9.7)	25 (80.6)	31
Zoukougbeu	49 (89.1)	39 (70.9)	35 (63.6)	0	53 (96.4)	55
Total	300 (78.5)	221 (57.8)	186 (48.7)	16 (4.2)	335 (87.7)	382

### Sample Selection for Variable Number Tandem Repeat Typing

A total of 243 confirmed BU samples were selected for VNTR typing. Out of the 243 BU cases, 86 were samples from Ghana from Amansie Central District (44), Ga West Municipal District (20), and Akuapim South Municipal District (22). For Côte d’Ivoire, 157 BU samples were selected from Bouaké (11), Divo (5), Kongouanou (27), Sakassou (15), Sinfra (3), Toumoudi (35), Yamoussoukro (28), and Zoukougbeu (33).

### *Mycobacterium ulcerans* DNA Confirmation From the Environment

**FIGURE 3:**
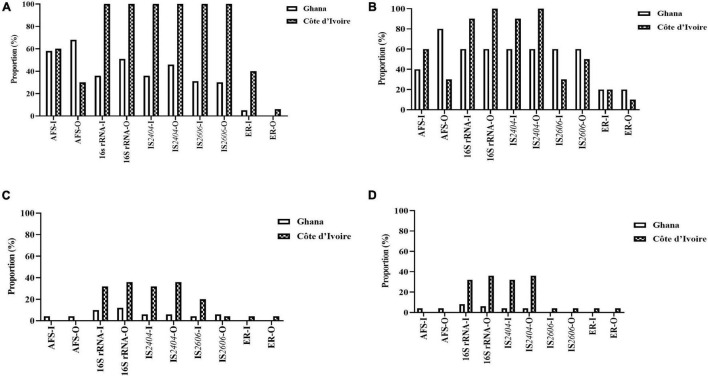
Positivity rate of the different detection methods of *M. ulcerans*, **(A)** water, **(B)** biofilm, **(C)** detritus, and **(D)** soil samples collected from Ghana and Cote d’Ivoire. I, inner section of the water body; O, riparian zone of the water body.

All environmental samples were first screened for the presence of acid-fast bacilli by microscopy, followed by PCR confirmation of NTM by 16S rRNA, *M. ulcerans* DNA by IS*2404*, and IS*2606* and a MPM by ER (Table 2B). Generally, a significantly higher proportion of *M. ulcerans* DNA was identified in the environmental matrices from Côte d’Ivoire compared with Ghana (chi-square for contingency table, *p* = 0.0031) (Figure 3).

**TABLE 2B:** *M. ulcerans* DNA positivity from environmental samples.

Location	Water (%)	Borehole (%)	Biofilm (%)	Soil (%)	Detritus (%)	Pond (%)
**Ghana**						
Amansie central	10/20 (50)	2/2 (100)	7 (35)	1/20 (5)	2/20 (10)	1 (0)
Akuapim south	1/3 (33.3)	3/3 (100)	6/10 (60)	2/10 (20)	4/10 (40)	0 (0)
**Côte d’Ivoire**						
Zoukougbeu	2/2 (100)	0 (0)	2/2 (100)	2/2 (100)	2/2 (100)	0 (0)
Kongouanou	4/4 (100)	0 (0)	4/4 (100)	4/4 (100)	4/4 (100)	0 (0)
Toumoudi	2/2 (100)	0 (0)	2/2 (100)	2/2 (100)	2/2 (100)	0 (0)
Sakassou	4/4 (100)	0 (0)	3/4 (75)	2/4 (50)	3/4 (75)	0 (0)
Djekanou	2/2 (100)	0 (0)	2/2 (100)	2/2 (100)	1/2 (50)	0 (0)
Bouake	2/2 (100)	0 (0)	2/2 (100)	1/2 (50)	2/2 (100)	0 (0)
Sinfra	2/2 (100)	0 (0)	2/2 (100)	2/2 (100)	1/2 (50)	0 (0)
Divo	2/2 (100)	0 (0)	2/2 (100)	2/2 (100)	1/2 (50)	0 (0)

*NB: M. ulcerans DNA positivity was determined via a positive PCR for IS2404 or an IS2606 and ER if negative for IS2404 or IS2606.*

### Confirmation and Comparison of *Mycobacterium ulcerans* DNA From Environmental Samples in Both Countries

#### Detection of *Mycobacterium ulcerans* DNA in Water Samples

*Mycobacterium ulcerans* DNA detection was higher in water bodies sampled from Côte d’Ivoire (100%) compared to Ghana (41%) with all the targeted genes amplified (*p* < 0.0001) (chi-square contingency table for independence). Identification using 16S rRNA (100% for Côte d’Ivoire; about 50% for Ghana) for Mycobacteria spp. was higher compared with acid-fast staining (AFS), *IS2404*, IS*2606*, and ER (Figure 3A). The lowest positivity rates were observed for MPMs when the ER domain was targeted (less than 40% for Côte d’Ivoire; about 10% for Ghana). Additionally, more *M. ulcerans* DNAs were detected in water samples collected from the riparian zone (O) compared with those collected from the water floor (I). In water sampled from boreholes, there was a 100% positivity rate of detection of acid-fast bacilli, 16S for mycobacteria, *IS2404*, and IS*2606*. However, there was no successful amplification of the ER gene in water samples albeit being positive for mycobacterial DNA using the other gene targets.

#### Detection of *Mycobacterium ulcerans* DNA in Biofilm From Aquatic Plants

In total, 50 biofilm samples from plants were collected from Amansie Central District (20), Akuapim South Municipal District (10), and Côte d’Ivoire (20). *M. ulcerans* DNA detection was higher in aquatic plants sampled from Côte d’Ivoire (96%) compared with that from Ghana (46%) with all the targeted genes amplified (*p* < 0.0003) (chi-square contingency table for independence). Positive amplification of the 16S rRNA gene and *IS2404* was higher compared with acid-fast bacilli detection and amplification of the *IS2606* and ER genes (Figure 3B). More *M. ulcerans* DNAs (based on the amplification of IS*2404* and IS*2606*) were detected among biofilms from the riparian zone (outer-O) compared with those collected from the water floor (inner-I). Amplification of 16Sr RNA and *IS2404* was higher in biofilm sampled from Côte d’Ivoire compared with that from Ghana. However, the detection of IS*2606* was found to be higher among the aquatic plants sampled from Ghana compared with those from Côte d’Ivoire. The proportion of ER and acid-fast bacilli detected in the riparian zone was higher among those from Ghana compared with those from Côte d’Ivoire. Differences in positivity rates based on sampling points were found to be statistically significant (*p* < 0.0001) when tested with the chi-square contingency table for independence.

#### Detection of *Mycobacterium ulcerans* DNA in Detritus

In total, 50 detritus material samples were obtained along the riparian zone and in the water body from Amansie Central District (20), Akuapim South Municipal District (10), and the communities in Côte d’Ivoire (20). Overall, a low detection rate of mycobacteria DNA was observed among the detritus materials, and *M. ulcerans* DNA was found to be higher among detritus materials sampled from Côte d’Ivoire (78%) compared with that from Ghana (25%). Again in this study, the highest positivity was found for the 16S rRNA gene for *Mycobacteria* spp. (about 40% for Côte d’Ivoire and less than 20% for Ghana) (Figure 3C). The lowest positivity was observed for ER for the detection of MPMs. The ER gene was detected in detritus samples from Côte d’Ivoire only (about 5%). Generally, more *M. ulcerans* DNAs (based on the amplification of IS*2404* and IS*2606*) were detected among samples collected from the riparian zone (outer-O) compared with those collected from the water floor (inner-I). No acid-fast bacilli were detected from microscopy among the detritus sampled.

#### Detection of *Mycobacterium ulcerans* DNA From Soil

In total, 50 soil samples were collected from both the riparian zone (outer-O) and in the river (inner-I) from Amansie Central District (20), Akuapim South Municipal District (10), and Côte d’Ivoire (20). Generally, *M. ulcerans* DNA detected from the soil was higher among samples taken from Côte d’Ivoire (87%) compared with those from Ghana (12%). The highest positivity was observed in the amplification of the 16S rRNA (about 35%). Generally, more *M. ulcerans* DNAs (based on the amplification of IS*2404* and IS2606) were detected among samples collected from the riparian zone (outer-O) compared with those collected from the river (inner-I). ER and IS*2606* genes were detected only in soil sampled from Côte d’Ivoire (Figure 3D). Acid-fast bacilli were detected only in the soils sampled from Amansie Central District, Ghana.

### Variable Number Tandem Repeat Typing of Clinical and Environmental Samples

A total of 6 MPMs consisting of *M. ulcerans 1615*, *Mycobacterium marinum hybrid 270995*, *M. marinum DL 180892*, *Mycobacterium pseudoshottsii*, *M. marinum CL*, and *M. marinum SA 2000695* (obtained from Mississippi State University) were genotyped to serve as standards for samples selected for VNTR profiling targeting 9 unique loci. The loci amplified in all DNA samples ranged from 1.1 to 86% with band sizes ranging from 200 to 900 bp. Two out of the ten loci targeted could not be amplified in any of the environmental samples (Locus 13 and Locus 33). There was amplification at Locus 6 and Locus 16 for a majority of the environmental samples, while MIRU 1 and STI 1 were the predominantly amplified loci for clinical samples (Tables 3A,B). Locus 13, Locus 33, and MIRU 9 were only rarely amplified in clinical samples.

**TABLE 3A:** Frequency distribution of successful amplification at the different loci for the clinical samples.

Locus	Ghana (%) *N* = 86	Côte d’Ivoire (%) *N* = 157	Total *N* = 243
MIRU 1	74 (86)	114 (72.6)	175 (77.4)
Locus 6	57 (66.3)	114 (72.6)	177 (72.8)
STI	63 (73.2)	114 (72.6)	177 (72.8)
Locus 19	58 (67.4)	89 (56.7)	147 (60.5)
Locus 15	52 (60.4)	90 (57.3)	142 (58.4)
Locus 18	48 (55.8)	17 (10.8)	65 (26.7)
Locus 13	13 (15.1)	2 (1.2)	15 (6.2)
Locus 33	13 (15.1)	4 (2.5)	17 (7.0)
MIRU 9	1 (1.1)	5 (3.1)	6 (2.5)

**TABLE 3B:** Frequency distribution of successful amplification at the different loci for the environmental samples.

LOCI	Ghana (%) *N* = 20	Côte d’Ivoire (%) *N* = 18	Total (%) *N* = 38
MIRU 1	15 (65)	12 (66.7)	27 (71)
Locus 6	15 (75)	12 (66.7)	27 (71)
STI	15 (75)	11 (61.1)	26 (68.4)
Locus 19	15 (75)	8 (44.4)	23 (60.5)
Locus 15	12 (60)	13 (72.2)	25 (65.8)
Locus 16	15 (75)	13 (72.2)	28 (73.7)
Locus 18	14 (70)	10 (55.6)	24 (63.1)
MIRU 9	13 (65)	12 (66.7)	25 (65.8)

#### Variable Number Tandem Repeat Profiles of Buruli Ulcer Clinical Samples Reveal New Genotypes Among the Two Countries

**FIGURE 4:**
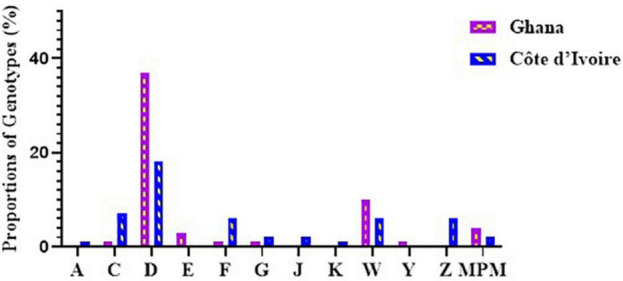
Diversity of *M. ulcerans* genotypes observed in clinical samples from Ghana and Côte d’Ivoire. Proportions were determined by the number of successfully designated VNTR genotypes out of the total number of confirmed BU clinical isolates used for VNTR typing.

Out of the 243 BU-positive clinical samples, 127 (52.3%) samples were successfully genotyped using at least the four standard loci, while 116 (47.7%) samples were characterized as indeterminate due to no amplification at one or more of the four standard loci. Genotypes were assigned based on published data from Williamson et al. (2014) and Narh et al. (2015), while samples that were different from already published data were assigned new genotypes. Eleven *M. ulcerans* VNTR genotypes, designated as A, C, D, E, F, G, Z, J, K, W, and Y (Table 4), were observed (Figure 4), and this was done to tally with previously identified genotypes. The four standard loci used (i.e., Locus 6, MIRU 1, ST1, and Locus 19) mainly gave variable repeats between 1 and 3 after sequencing. Majority of the genotypes observed were Genotype D (25.1%). This was followed by Genotype W (7.4%), Genotype C (5%), Genotype F (4.2%), Genotype Z (3.9%), Genotypes G and J (1.5%), Genotype Y (1.1%), and, finally, Genotype A and Genotype K (0.8% each).

**TABLE 4:** Designated genotypes identified with reference studies as standard.

	VNTR profiles				
Designated genotypes	MIRU 1	Locus 6	ST1	Locus 19	Reference (sample size)
A (MU)	1	1	1	2	Current study (159)
C (MU)	3	1	2	2	
D (MU)	1	1	2	2	
E (MU)	1	1	2	3	
F (MU)	2	1	2	2	
G (MU)	2	1	2	1	
Z (MU)	1	2	2	2	
J (MU)	2	2	2	1	
K (MU)	2	2	2	2	
MLF (MPM)/Y	1	2	2	1	
MSA	1	3	3	1	
MCL (MPM)	1	2	2	3	
MDL (MPM)	1	3	2	1	
MHB (MPM)	1	3	2	1	
W	1	1	2	1	Narh et al., 2015 (15)
X	1	1	2	2	
Y	1	2	2	1	
Z	1	2	2	2	
A	1	1	1		Williamson et al., 2008 (50)
B	3	1	1		
C	3	1	2		
D (unpublished strain)	1	1	2	2	
MMDL	1	4	2	2	
	1	2	1	2	
MLF	1	2	2	1	
MPS	1	4	2	2	

*MDDL, NB: M. marinum DL 180892; MLF, M. liflandi, MPS, M. pseudoshotii.*

In Ghana, 7 *M. ulcerans* genotypes were observed. Majority were genotype D (37.2%). The rest were Genotypes W (9.6%) and E (3.2%), with the least observed as Genotypes C, F, G, and Y (1.1%) each. Other MPMs were identified in 4.2%, while 41.5% were indeterminate. The least variation among the genotypes was observed in Ga East District (Genotypes D and W), followed by Amansie Central District (Genotypes D, E, and W), with the most diverse genotype observed in Akuapim South District (Genotypes C, D, F, G, W, and Y). In Côte d’Ivoire, more diverse genotypes of *M. ulcerans* were observed. Nine *M. ulcerans* genotypes were identified with the majority being Genotype D (18.1%), followed by Genotype C (7.2%), Genotypes F, Z, and W (6.0%), Genotype J (2.4%), Genotype G (1.8%), Genotype K (1.2%), with the least being Genotype A (0.6%). A total of 77 (46.3%) isolates were indeterminate due to no amplification at one or more of the four standard loci. The most diverse genotypes were observed in Zoukougbeu (Genotypes C, D, G, Z, J, W, and Y) and Kongouanou (Genotypes C, D, F, Z, J, and W) with the least in Sinfra (Genotypes D and W). Genotype E was observed only among the Ghanaian samples, while Genotypes A, Z, J, and K were observed only among the Ivorian samples (Figure 4).

#### Variable Number Tandem Repeat Typing of Environmental Isolates Reveals More Conserved but Similar Genotypes Between the Two Countries

**FIGURE 5:**
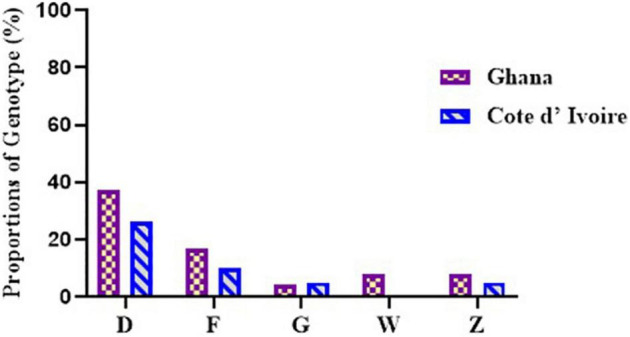
Diversity of *M. ulcerans* genotypes observed among the environmental isolates from Ghana and Côte d’Ivoire. Proportions were determined by the number of successfully designated VNTR genotypes out of the total number of confirmed BU environmental isolates used for VNTR typing.

A total of 5 *M. ulcerans* genotypes, designated as D, F, Z, G, and W, were observed in environmental isolates (Figure 5). The four standard loci used mainly gave variable repeats of 1 and 2. Majority, 161/204 (78.9%), of the DNA from the environmental samples failed to be amplified at any of the loci used for this study. At least one or more loci were amplified in DNA from the other 43 samples. Majority of the genotypes observed were Genotype D (32.5%), followed by Genotype F (13.9%), Genotype Z (7.0%), and finally Genotype G and Genotype W (4.6% each). A total of 37.2% of genotypes were indeterminate as amplification could not be achieved in all the four standard loci.

In environmental samples collected from Côte d’Ivoire, Genotype D (26.3%), Genotype F (10.5%), and Genotypes G and Z (5.2%) were observed. Notably, 10 genotypes (52.6%) were indeterminate due to no amplification in one of the four standard loci. Genotype D, Genotype F, and Genotype G were observed among DNA from the water samples, Genotypes F and Z were observed among the biofilms from aquatic plants, and Genotype D was observed from DNA obtained from the soil. None of the isolates obtained from detritus samples could be genotyped due to no amplification in one or more of the standard loci. However, five genotypes were observed in the environmental samples from Ghana, namely, Genotype D (37.5%), Genotype F (16.7%), Genotype Z and Genotype W (8.3% each), and Genotype G (4.1%). A total of 6 genotypes (25.0%) were indeterminate due to no amplification at one or more of the four standard loci. Four genotypes were observed from boreholes (Genotypes D, F, G, and W), three from rivers (Genotypes D, F, and Z), two from soil samples (Genotypes D and W), and one from detritus samples (Genotype D). No genotype was designated for biofilm samples obtained from aquatic plants as there was no amplification in one or more of the standard loci. Comparing environmental isolates from Ghana and Côte d’Ivoire showed that Genotype W was observed only among the Ghana environmental samples.

### Clinical and Environmental Genotypes Show Similar Circulating Mycolactone-Producing Mycobacteria Strains

In total, 11 MU genotypes (Genotypes A, C, D, E, F, G, Z, J, K, W, and Y) were identified in this study from human and environmental samples (Table 4). Discrimination using the four VNTR standard markers (i.e., Locus 6, ST1, MIRU 1, and Locus 19) revealed evolving heterogeneity of *M. ulcerans* between the two countries. Comparing our findings with other published genotypes shows conserved repeats of 1 and 2 for MIRU 1 and ST1, respectively. Locus 6 and Locus 19 had evolved with discriminatory repeats of 3, besides the historical repeats of 1 and 2. This led to the designation of new genotypes, Genotype E, Genotype F, Genotype G, Genotype J, and Genotype K, in this study. Of note, 5 out of the 11 genotypes (Genotypes D, F, G, Z, and W) obtained in the clinical samples were identical to those obtained from the environment. Genotype D was predominant in both the clinical and environmental samples from both Ghana and Côte d’Ivoire. Comparing environmental samples with clinical samples from Ghana, Genotype Z was unique among the environmental samples, while Genotypes C, E, G, and Y were found only in clinical samples. In Côte d’Ivoire, no genotype was found to be unique among the environmental isolates. However, a greater diversity existed in genotypes among the clinical samples compared with the environmental samples with unique genotypes, Genotypes A, C, J, W, and K, identified only in the clinical samples.

## Discussion

Buruli ulcer has been found to be prevalent in localized communities in Ghana and in most rural communities in Côte d’Ivoire (Williamson et al., 2008; Ablordey et al., 2015; Dassi et al., 2017). However, there has been less comparison between the epidemiology of MPMs obtained from the environments and clinical sources in these 2 countries. This study is the first to explore and compare clinical and environmental genotypes of *M. ulcerans* from two BU endemic countries, namely, Ghana and Côte d’Ivoire. It also explored the similarity between *M. ulcerans* genotypes from the environment and those causing human infections.

Association with an aquatic environment has been reported to be the main environmental risk factor for BU transmission (Williamson et al., 2008). In this study, we explored the distribution of MPMs in BU endemic environments in Ghana and Côte d’Ivoire. A similar study conducted in Benin identified an association between the presence of *M. ulcerans* DNA in the environment and the number of BU cases (Williamson et al., 2012).

The water (surface water and wells) sampled for this study was mainly used for the purpose of farming, fishing, and recreational activities mainly by children. Generally, a higher detection rate of *M. ulcerans* DNA was found in water sampled from Côte d’Ivoire compared with that from Ghana. *M. ulcerans* DNA was found to be equally present in both riparian zone and the water floor. A higher detection was observed in water sampled at the banks (riparian zone) compared with the inner section of the rivers. Frequent usage and exposure to these water bodies, especially at the riparian zones, could be a source of *M. ulcerans* exposure to individuals in these communities. A higher amplification of the ER domain gene (involved in mycolactone synthesis) in the water sampled from Côte d’Ivoire compared with Ghana could explain the higher prevalence of BU in Côte d’Ivoire compared with Ghana (Williamson et al., 2008, 2012; Narh et al., 2015). The prevalence of mycobacteria in all boreholes sampled is alarming, since borehole water is mainly used for domestic purpose, such as cooking, bathing, and drinking. However, the inability to detect the ER domain gene in borehole water could suggest the absence of *M. ulcerans* plasmid needed for mycolactone synthesis (Qi et al., 2009; Nakanaga et al., 2018). Further studies on *M. ulcerans* identification in boreholes in BU endemic communities are recommended.

The prevalence of mycobacteria identified in the biofilm was higher in Côte d’Ivoire compared with Ghana. Even though other studies found a higher prevalence of MU DNA in biofilms from aquatic plants (Williamson et al., 2008; Williamson et al., 2014; Narh et al., 2015), this study comparatively found a higher prevalence in the rivers as found in a similar study conducted in Cameroun (Zeukeng et al., 2021). There was also a higher proportion of the ER domain gene amplification in biofilm from aquatic plants compared with those found in open water in Ghana and Côte d’Ivoire. These findings corroborate aquatic plants as possible reservoirs for *M. ulcerans* and risk factors in BU transmission in Ghana and the preferred ecological niche for both countries as described in previous studies (Williamson et al., 2012; Narh et al., 2015).

The aquatic environmental matrix with the least prevalence of MPMs was the soil in Ghana and the detritus material in Côte d’Ivoire. Generally, a low amount of MPMs were detected in soil and detritus samples in both countries with a higher proportion at the banks of the water bodies compared with those sampled in the water floor. The ER domain gene could not be detected in both soil and detritus in Ghana, while a low proportion was detected in Côte d’Ivoire. Soil and detritus may thus be unlikely reservoirs for *M. ulcerans* transmission in Ghana. Detection of the ER domain in soil and detritus samples from Côte d’Ivoire suggests a possible, but low, potential reservoir for *M. ulcerans* transmission in Côte d’Ivoire. Even though there was low detection of *M. ulcerans* DNA among the detritus and soil samples in both countries in the riparian zone and in the rivers, a previous study indicated that *M. ulcerans* DNAs are likely to remain in underwater decaying organic matter (Bratschi et al., 2014).

This study has also confirmed the need for the application of different confirmatory or detection tools for MPM in both clinical and environmental samples (Fyfe et al., 2007; Yeboah-Manu et al., 2018). Even though detection of IS*2404* remains the gold standard for *M. ulcerans* identification in clinical samples, this insertion sequence has been detected in MPMs other than *M. ulcerans*. Amplification of the ER domain gene and VNTR typing will help to differentiate between *M. ulcerans*/MPMs and mycobacteria present in the environment and BU wounds. A higher prevalence of IS*2404* detection compared with ER in both countries was observed with water sampled from boreholes and may likely reflect the presence of other mycobacterial species harboring the insertion sequences IS*2404* and IS2606, which have not yet been confirmed to cause BU in humans (Williamson et al., 2012; Narh et al., 2015; Dassi et al., 2017).

Even though a greater discrimination between *M. ulcerans* could be achieved by amplification of more than the four standard loci (i.e., MIRU 1, Locus 6, ST1, and Locus 19) for VNTR typing, our finding suggests that determining heterogeneity between MPMs from different countries can still be achieved with the four standard loci. Apart from the four standard loci, majority of the loci amplification were obtained at Locus 18 and Locus 16 for the environmental samples and at Locus 15 for the clinical samples. However, they did not have much discriminatory power to determine heterogeneity between *M. ulcerans* (two repeats for Locus 18, one repeat for Locus 16 and Locus 15). Nevertheless, Locus 15 and Locus 33 helped in the differentiation of *M. ulcerans* from other MPMs. These loci could be ideal as another genotypic tool in differentiating *M. ulcerans* from other MPMs. Repeats of one and two for Locus 6 and ST1 from this study are consistent with previous studies (Ablordey et al., 2005; Hilty et al., 2006; Narh et al., 2015). Repeats of one and three at MIRU 1 have also been reported (Williamson et al., 2014), which was consistent with this study.

In this study, genotyping *M. ulcerans* samples were done in clinical samples from both countries and then compared with genotypes of environmental samples collected from both countries. This enabled matching common genotypes from clinical and environmental sources from both countries. All samples obtained were classified into 11 *M. ulcerans* genotypes using already published data as references (Williamson et al., 2014; Narh et al., 2015). However, repeats of two and three at MIRU 1 and Locus 19 helped in the detection of new genotypes circulating both in the environment and in patients in the two countries. Out of the 11 genotypes observed, at least five circulating genotypes (Genotypes C, D, F, G, and W) were common among the clinical samples from both countries. Genotype D was found to be the predominant genotype in circulation in both countries and was found at each sampling site.

Unique genotypes were identified in both countries with Genotypes A, Z, J, and K unique to Côte d’Ivoire and Genotypes E and Y unique to Ghana. Among the three districts in Ghana where clinical samples were taken, the Akuapem South District had the most diverse genotypes. Genotypes F, G, and Y, as well as other MPMs, were identified in this district. This suggests a more diverse heterogeneity among *M. ulcerans* from this district compared with the others. Genotypes E and W were found only in the Amansie Central District. The least diverse genotype distribution was observed in the Ga-West District (Genotype D and Genotype W). These findings suggest that environmental factors such as geographical location may affect the distribution of *M. ulcerans* genotypes (Stragier et al., 2005). In Côte d’Ivoire, among the eight sites sampled, Konguouanou and Zoukougbeu had the most diverse genotypes (six genotypes each). Genotype K was identified in only Yamoussoukro, while Genotype Y previously identified in Amansie Central District in Ghana (Narh et al., 2015) was identified in Zoukougbeu. Even though *M. ulcerans* genotypes were quite similar in the two countries, detection of other unpublished genotypes unique to each country suggests the possibility of genetic differences existing in *M. ulcerans* in the two countries.

Variations among the environmental samples from both countries were limited. Only 5 genotypes were identified (Genotypes D, F, G, Z, and W). Both countries had the same genotypes for the environmental samples with the exception of Genotype W, which was only identified in Ghana. Genotype D was predominant in both countries at all sampling sites. Studies from 2008 till date (Narh et al., 2015). Williamson et al. (2008), Williamson et al. (2014) show that Genotypes D, C, W, and Y have persisted within the BU environment and in clinical lesions. Nonetheless, Genotype D was found in almost all the clinical and environmental sites used for this study and may possibly suggest the first *M. ulcerans* genotype in circulation from which other genotypes evolved. Evolutionary studies of *M. ulcerans* genotype will be needed for this clarification. Further studies are also needed to identify key factors that may play a role in the dominance of Genotype D in both clinical and environmental samples. However, the identification of new strains, particularly in BU lesions, suggests the possibility of *M. ulcerans* evolving to adapt to their new environments.

Even though the mode of *M. ulcerans* transmission remains unknown, several postulations have been proposed, which include inoculation into open wounds of exposed skin (Raghunathan et al., 2005). Activities such as swimming and bathing and agricultural activities such as farming and fishing in *M. ulcerans*-contaminated rivers or ponds may expose an individual to *M. ulcerans*. Individuals could be inoculated through exposure to biofilms on aquatic plants, the soil, or water bodies where there is a cut or abrasion on the skin (Williamson et al., 2014; Wallace et al., 2017).

## Conclusion

Based on VNTR genotyping on *M. ulcerans* isolates, 11 genotypes were identified among the clinical isolates, while 6 were identified from the environmental isolates in both countries. Clinical *M. ulcerans* Genotypes C, D, F, and G were common in both countries. Diverse genotypes were observed among the clinical isolates in Côte d’Ivoire compared with that in Ghana. For the environmental samples, common genotypes were observed in both countries except Genotype W, which was observed among the Ghanaian environmental samples. Genotype D was found to be prominent in all the sampling locations of both clinical and environmental samples. Common genotypes observed in both environmental and clinical samples suggest possible transmission of *M. ulcerans* from the environment, particularly water bodies and biofilms from aquatic plants, to human through open lesions on the skin.

## Data Availability Statement

The datasets presented in this study can be found in online repositories. The names of the repository/repositories and accession number(s) can be found at https://www.ncbi.nlm.nih.gov/sra/PRJNA812065.

## Ethics Statement

The studies involving human participants were reviewed and approved by the Institutional Review Board of Noguchi Memorial Institute for Medical Research (052/17-18), Ghana Health Service Ethical Review Committee (GHS-ERC017/07/17), Comité National d’Ethique et de la Recherche (CNER) in Abidjan, Côte d’Ivoire (112-18/MSHP/CNESVS-km). Written informed consent to participate in this study was provided by the participants’ legal guardian/next of kin.

## Author Contributions

LM and CQ supervised the study and reviewed the manuscript for intellectual content. EG designed the study, conducted the laboratory and data analysis, and drafted the manuscript. MS-D participated in the laboratory and data analysis and assisted with the drafting of the manuscript. AA coordinated with the sample collection in Côte d’ Ivoire. MS-D, EK-B, and DA-A assisted with laboratory and field work. All authors read and approved the final version of the manuscript.

## Author Disclaimer

The views expressed in this publication are those of the author(s) and not those of the funding agency.

## Conflict of Interest

The authors declare that the research was conducted in the absence of any commercial or financial relationships that could be construed as a potential conflict of interest.

## Publisher’s Note

All claims expressed in this article are solely those of the authors and do not necessarily represent those of their affiliated organizations, or those of the publisher, the editors and the reviewers. Any product that may be evaluated in this article, or claim that may be made by its manufacturer, is not guaranteed or endorsed by the publisher.
